# Polyautoimmunity Reflecting Immune Dysregulation in Common Variable Immunodeficiency

**DOI:** 10.3390/biomedicines13030552

**Published:** 2025-02-21

**Authors:** Maria Giovanna Danieli, Giuseppe Murdaca, Cristina Mezzanotte, Ilaria Claudi, Elena Buti, Matteo Martini, Maria Beatrice Bilò, Sebastiano Gangemi, Gianluca Moroncini

**Affiliations:** 1 SOS Immunologia delle Malattie Rare e dei Trapianti, SOD Clinica Medica, Dipartimento di Medicina Interna, Azienda Ospedaliero Universitaria delle Marche, 60126 Ancona, Italy; m.g.danieli@univpm.it; 2 Department of Clinical and Molecular Sciences, Marche Polytechnic University, 60126 Ancona, Italy; m.b.bilo@univpm.it (M.B.B.); g.moroncini@univpm.it (G.M.); 3 Postgraduate School in Allergology and Clinical Immunology, Università Politecnica delle Marche, 60126 Ancona, Italy; ilari.claudi@gmail.com (I.C.); elena.buti2017@gmail.com (E.B.); 4 Department of Internal Medicine, University of Genoa, 16132 Genoa, Italy; 5 Allergology and Clinical Immunology Unit, Ospedale San Bartolomeo, 19038 Sarzana, Italy; 6 UO Medicina Interna, Azienda Sanitaria Territoriale Pesaro Urbino, 61032 Fano, Italy; cristina.mezzanotte.1@gmail.com; 7 SOSD Allergologia, Dipartimento di Medicina Interna, Azienda Ospedaliero Universitaria delle Marche, 60126 Ancona, Italy; matteo.martini@ospedaliriuniti.marche.it; 8 Operative Unit of Allergy and Clinical Immunology, Department of Clinical and Experimental Medicine, University of Messina, Via Consolare Valeria 1, 98125 Messina, Italy; sebastiano.gangemi@unime.it; 9 Clinica Medica, Department of Internal Medicine, Marche University Hospital, 60126 Ancona, Italy

**Keywords:** antinuclear antibodies, autoantibodies, autoimmune disease, autoimmunity, common variable immunodeficiency, immune dysregulation, immune imbalance, inborn errors of immunity, intravenous immunoglobulin, polyautoimmunity, subcutaneous immunoglobulin

## Abstract

**Background**: Common variable immunodeficiency (CVID) is the most frequent symptomatic inborn error of immunity (IEI) in adulthood. Other than recurrent infections, CVID may present with non-infectious complications such as enteropathy, lymphoproliferation, malignancy, and autoimmune diseases. Patients could have a single autoimmune disease (monoautoimmunity) or two or more autoimmune diseases (overt polyautoimmunity). “Latent polyautoimmunity” corresponds to the presence of autoantibodies without a clinically evident autoimmune disease. **Methods**: The aim of this retrospective study was to describe autoimmunity and polyautoimmunity in a population of 81 CVID adult patients, enrolled from January 2008 to July 2022 (mean follow-up: 8.5 years). **Results**: We documented at least one autoimmune disorder in 40 patients (49.4%). Moreover, 15 subjects (37.5% of patients with autoimmunity and 18.5% of all CVID population) presented polyautoimmunity. Despite the humoral immune deficiency, we detected different autoantibodies in CVID patients with or without a concomitant autoimmune disease. In both groups with monoautoimmunity and polyautoimmunity, cytopenias were the most common manifestation. Conversely, enteropathy was recorded only in patients with polyautoimmunity (27%, *p* = 0.006). Patients with polyautoimmunity showed a significantly lower mean age at diagnosis (−12 years, *p* = 0.018) compared to those with monoautoimmunity. We documented a higher frequency of autoimmunity in CVID patients who had increased diagnostic (+5.6 years) and therapeutic (+7.2 years) delay (*p* = 0.093 and 0.054, respectively). **Conclusions**: Polyautoimmunity is a frequent condition in patients affected by CVID. An early start of Ig replacement therapy could help prevent autoimmune complications.

## 1. Introduction

Common variable immunodeficiency (CVID) is the most common symptomatic inborn error of immunity (IEI) in adulthood, characterized by reduced serum levels of immunoglobulin (Ig) and impaired specific antibody production [1].

Other than recurrent infections, CVID includes a wide range of clinical manifestations, such as inflammatory and autoimmune diseases, granulomatosis, lymphoproliferation, enteropathy, allergy, and malignancy [2]. Due to the great variability of clinical manifestations, the diagnosis of CVID is often delayed, causing significant morbidity and mortality. Each year of diagnostic delay is associated with an increased risk of death, bronchiectasis, solid tumors, and enteropathy [3].

Immune dysregulation and the resulting breakdown of immune tolerance in CVID are responsible for various autoimmune diseases, which may represent the first manifestations of this disorder. They occur in a percentage between 21–42% in CVID and approximately in 30% of patients with other IEIs [3,4,5,6]. Most, if not all, autoimmune or immune-mediated diseases are described in patients with CVID, ranging from gastrointestinal to rheumatological, dermatological, endocrine, and neurological ones [5]. The most common autoimmune complications are autoimmune cytopenias (mainly immune thrombocytopenia, ITP), detected in 11–18% of CVID patients. In CVID patients with autoimmune diseases, other frequently reported non-infectious complications are lung disease, granulomatosis, lymphoproliferation, lymphomas, liver disease, and enteropathy [7,8,9].

The coexistence of two or more autoimmune diseases fulfilling classification criteria in the same patients (“overt polyautoimmunity”) has been described in the last few years [10]. Moreover, subjects showing positivity for some autoantibodies in the absence of the corresponding autoimmune disease represent an interesting new category, termed “latent polyautoimmunity” [11]. Our work aimed to analyze the characteristics of CVID patients with autoimmunity and polyautoimmunity.

## 2. Patients and Methods

### 2.1. Setting and Patients

This retrospective study included adult patients, enrolled from January 2008 to July 2022, with a follow-up of at least 2 years, who were diagnosed with CVID based on the revised criteria established by the ESID (available at https://esid.org/working-parties/registry-working-party/diagnosis-criteria/, accessed on 19 February 2025) and/or the International Consensus Document (ICON) criteria for cases prior to the ESID criteria [1,12]. Of the patients who were diagnosed according to the ICON diagnostic criteria, we included in this study only those who presented during follow-up low serum IgA levels (<2 SD of the normal range for age), in addition to the initial low serum IgG and IgM levels. The CVID-associated complications followed the categorization outlined by Chapel et al. [13].

Diagnostic delay was defined as the time between the occurrence of the first CVID-related symptom (e.g., infection recurrences, ITP, neoplasia) and the effective CVID diagnosis. Therapeutic delay was defined as the time between the occurrence of the first CVID-related symptom and the initiation of Ig replacement therapy [14,15].

Comprehensive data collection performed for each patient, routine analyses, immunological parameters, and other exams are reported in Supplementary Materials.

### 2.2. Statistical Analysis

All variables of interest are presented as absolute frequencies and percentages for categorical data and as mean ± standard deviation (SD) or median with interquartile range [IQR] for normal or non-normal distributed continuous data, respectively. To compare clinical and demographic variables between patients with and without autoimmune diseases, the *t*-test (after log-transformation in case of non-normal distributions) and chi-squared test were utilized, as appropriate. Subgroup analyses were performed according to different categories of age of onset of symptoms and age of diagnosis (10–40, >40–60, and >60 years old). Assessment for normal distribution was graphically performed. The database was locked on 16 July 2024 for the statistical analysis, which was performed using SPSS software (SPSS version 21.0 IBM, Armonk, NY, USA).

## 3. Results

### 3.1. Baseline Characteristics

Table 1 details the main characteristics of our series of 81 CVID patients followed up over a mean period of 8.5 years.

### 3.2. Autoimmune Manifestations

**Figure 1:**
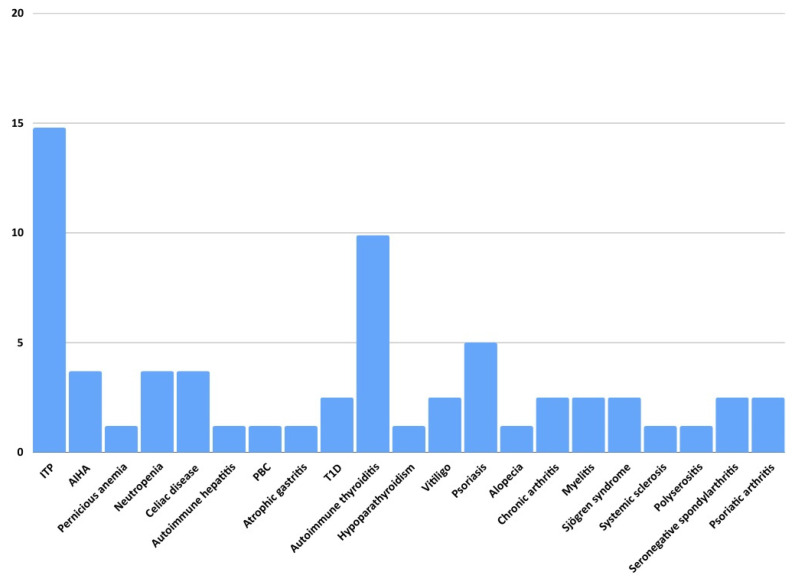
Prevalence of autoimmune manifestations in our cohort of 81 CVID patients. Abbreviations: AIHA: autoimmune hemolytic anemia, ITP: immune thrombocytopenia, PBC: primary biliary cholangitis, T1D: type 1 diabetes.

**Figure 2:**
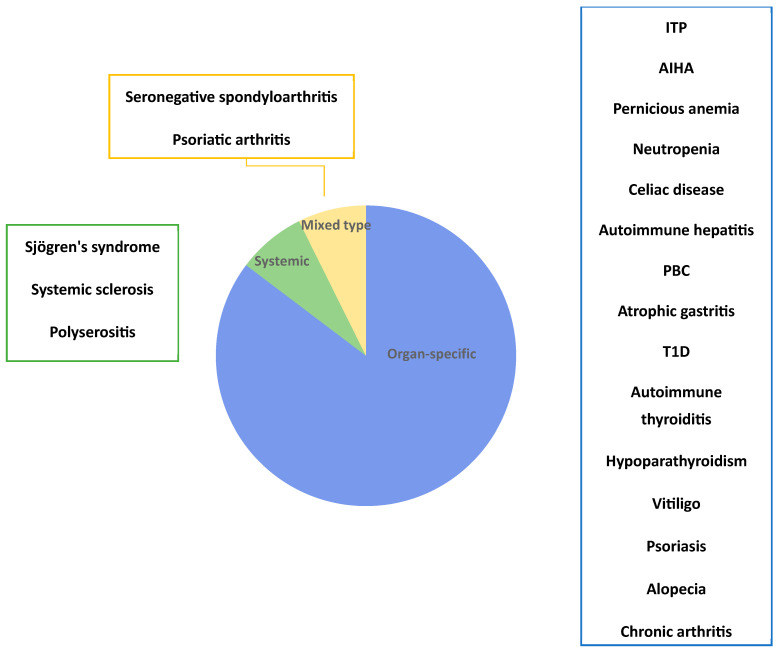
Prevalence of organ-specific, systemic, or mixed type autoimmune diseases in our cohort of 81 CVID patients. Abbreviations: AIHA: autoimmune hemolytic anemia, ITP: immune thrombocytopenia, PBC: primary biliary cholangitis, T1D: type 1 diabetes.

In our series, 40/81 patients (49%) developed at least one autoimmune disease. They were 32 females (80%), with a mean ± SD age at CVID diagnosis of 44 ± 16 years old. Their main characteristics are reported in Table 2. The autoimmune diseases presented in our series were heterogeneous and involved different organ systems, as shown in Figure 1 and Figure 2. Cytopenias were the most common and were present in 19 patients (16 females, 84%) and were mainly represented by ITP (*n* = 12). Eleven patients had an endocrine autoimmune disease with predominant autoimmune thyroiditis (*n* = 8; 6 females, 75%) (Figure 1).

In our series, an autoimmune disease developed as a mean 6.2 years after CVID diagnosis. A single case showed that the autoimmune disease onset was 3 years prior to the clinical presentation and diagnosis of CVID. Other patients presented with an autoimmune disease at the onset and diagnosis of CVID, associated with recurrent infections that facilitated the diagnosis of CVID.

### 3.3. Comparison Between Patients with and Without Autoimmunity

Comparing the two groups of CVID patients with and without autoimmunity, we found that there were no significant differences in age and mean serum IgG levels at the diagnosis of CVID (Table 2). No difference related to the presence of autoimmunity was detected between pediatric and adult-onset patients (*p* = 0.949). Among the 20 pediatric onset patients, 10 (50%) had an autoimmune disease. In the group of 61 adult-onset patients, 30 (49%) had an autoimmune disease.

A trend toward a longer diagnostic (+5.6 years) and therapeutic (+7.2 years) delay was observed in patients with autoimmune diseases, even if the difference did not reach the threshold of statistical significance (*p* = 0.093 and 0.054, respectively) (Table 2). Conversely, significant differences were observed, among the different age of diagnosis subgroups, for both the diagnostic (*p* = 0.020) and therapeutic (*p* = 0.046) delays, but only within patients with autoimmunity. Significant differences were observed also among the age of symptoms’ onset subgroups, within the autoimmunity patients, for both diagnostic (*p* = 0.0144) and therapeutic (*p* = 0.0069) delays (Table 2).

Polyclonal lymphoproliferation and neoplastic complications were more prevalent in patients with autoimmune diseases in our cohort (+11% at *p* = 0.296 and +23% at *p* = 0019, respectively), while enteropathy was less common in patients without an autoimmune disease compared with patients with autoimmunity (−12%, *p* = 0.143) (Table 2).

### 3.4. Patients with Polyautoimmunity

Fifteen subjects (37.5% of patients with autoimmunity and 18.5% of all CVID population) presented polyautoimmunity. Their main characteristics are summarized in Table 3 and Table 4. Of those, 13 patients had two or more autoimmune diseases (overt polyautoimmunity). Two other subjects had latent autoimmunity due to the presence of autoantibodies in the absence of clinical symptoms or classification criteria for autoimmune diseases.

Regarding the 15 patients affected by polyautoimmunity (11 females, 73%), ITP (*n* = 6) and autoimmune thyroiditis (*n* = 5) were the prevalent manifestations. The most frequent CVID-related comorbidities were malignancies (*n* = 6, 40%), followed by lymphoproliferative disorders (*n* = 5, 33%), enteropathies (*n* = 4, 27%) and liver diseases (*n* = 2, 13%).

The comparison between patients with monoautoimmunity and polyautoimmunity, is shown in Table 5. In both groups, autoimmune cytopenias were the most common manifestation. Conversely, enteropathy was recorded only in patients with polyautoimmunity (27%, *p* = 0.006). Patients with polyautoimmunity showed a significantly lower mean age at diagnosis (−12 years, *p* = 0.018) compared to those with monoautoimmunity. Furthermore, polyautoimmunity was more frequently associated with age of diagnosis between 10 and 40 years old (64.7% of patients), compared to older age categories; conversely, patients with monoautoimmunity were less frequently associated with an early diagnosis between 10 and 40 years old (35.3% of patients) (*p* = 0.004). No significant differences emerged between the two groups concerning the other characteristics or subgroups.

Among 15 patients affected by polyautoimmunity, 7 (46.6%) patients were treated with IVIg, 4 (26.6%) with 20%SCIg and 3 (20%) with fSCIg at 0.4–0.6 g/kg/month; a subject was not treated with Ig due to personal choice. Patients with autoimmune cytopenias were successfully treated with Ig replacement therapy (intravenously or subcutaneously, at 0.5–0.6 g/kg/month) in combination with corticosteroid and immunosuppressive drugs. Three patients with ITP have been treated with facilitated SCIg (20 g twice a month for 6 months at the dosage of 0.6 g/kg/month). One patient affected by autoimmune hepatitis was treated with azathioprine for a short period and then suspended due to gastrointestinal intolerance. Patients with rheumatic conditions received medium-low doses of oral glucocorticoids (≤10 mg/day prednisone equivalent), hydroxychloroquine (200–400 mg/day), methotrexate (7.5 mg/week s.c.) or adalimumab (40 mg every 2 weeks s.c.). All patients tolerated these treatments well.

### 3.5. Autoantibodies in Autoimmunity and Polyautoimmunity

Serological investigations reveal the presence of autoantibodies in patients with CVID and an associated autoimmune disease. Five individuals were found to have ANA positivity, including four who had a single autoimmune disease (ANA with nucleolar pattern in the patient with autoimmune hemolytic anemia; ANA with homogenous pattern in both patients with Sjogren’s syndrome, and anti-centromere antibodies in the patient with systemic sclerosis). One patient with recurrent myelitis had anti-SSA antibodies. We also registered anti-TPO antibodies in eight patients: three patients with single autoimmunity (autoimmune thyroiditis, Sjӧgren’s syndrome, and systemic sclerosis), four patients with polyautoimmunity (three cases of thyroiditis associated with ITP, and one patient suffering from thyroiditis, vitiligo, and seronegative spondyloarthritis). One patient presented latent polyautoimmunity, with his mother affected by thyroiditis.

In our series, we noticed two cases of LAC positivity; the first patient suffered from ITP, and the second one did not develop an overt autoimmune disease, even if he presented some clinical features of Behçet’s disease without fulfilling diagnostic criteria (“Behçet minus” syndrome). These two patients who were positive for LAC did not experience any vascular or thrombotic event.

The two patients with latent autoimmunity who have positive anti-TPO antibodies and LAC have been monitored for 9 and 6 years, respectively, without developing clinical symptoms or meeting the classification criteria for a definite autoimmune disease.

## 4. Discussion

### 4.1. Autoimmune Diseases in CVID

We present our data related to autoimmunity and polyautoimmunity in patients with CVID. In our series, we registered a higher percentage of autoimmunity (49%) compared to 21–42% of the cases previously reported in the literature [3,4,5,6].

This higher percentage of CVID patients developing autoimmune diseases compared with previous reports can be explained by several factors. First, we are a tertiary referral center mainly dedicated to autoimmune diseases, so our diagnostic protocols always foresee the search for an autoimmune disease in our patients. We noticed that our patients have fewer respiratory complications such as bronchiectasis, so patients with a predominant “infection only” phenotype may be followed in other medical departments. Second, our population is mainly composed of female patients (66% of all patients). In our series, the most frequent autoimmune diseases were autoimmune cytopenias (84% females) and autoimmune thyroiditis (75% females). Overall, in the polyautoimmunity group, 11 were females (73%). The increased frequency of autoimmunity in our cohort may be due to the higher percentage of female patients since autoimmunity prevails in females [16,17]. Third, in our case series, the mean age at diagnosis was 44 years old, which is higher compared to ESID Registry data that reported a mean age at CVID diagnosis of 31 years old [3]. From a theoretical point of view, the probability of developing an autoimmune disease may increase with aging, i. due to the senescence of the immune system and the consequent loss of immune tolerance [15,18]; ii. the continuous exposure over the years to pathogens, different stressors, and environmental factors (i.e., exposome) that can contribute to a proinflammatory environment facilitating the emergence of an autoimmune disease [19]. Finally, another explanation could be linked to the huge increase in autoimmune diseases in the general population in the last years, as outlined by the works of Miller et al. [20,21]. According to the data of ESID Registry [3], the prevalence of all autoimmune diseases is 7.6 times higher in CVID patients compared to the general population. Despite significant differences in the geographical distribution of autoimmune diseases and variable frequencies of specific systemic autoimmune diseases in some populations, the increase in autoimmune diseases is real [16,17].

Concerning specific autoimmune disorders, in our and other series, ITP and autoimmune hemolytic anemia are the most common associated conditions, resulting 702.9 times more frequent in CVID patients than in the general population [3,22]. As in Feuille et al. [5], our patients with autoimmune cytopenias are significantly likely to have one or more other CVID-associated non-infectious complications, such as neoplasia. In line with our experience, it was found that patients with ITP have a 3-fold higher risk of developing cancer than those without ITP [14,23].

Rheumatological manifestations are detected in 11% of all our CVID patients (22.5% of the AD population). In agreement with the literature, rheumatologic diseases are reported in a range from 5.9% [24] to 10% of CVID patients [5], with, as expected, a female preponderance [24]. Psoriatic arthritis and cutaneous psoriasis are relatively frequent in our cohort, probably due to the higher prevalence of these conditions in our Region (3.5% in the Marche Region against 2.8% in Italy).

Regarding organ-specific autoimmune manifestations, our data agree with those of Odnolektova et al. [3], which showed a higher prevalence of hypothyroidism (3.5%), followed by alopecia areata (2.7%) and type 1 diabetes (1.6%).

### 4.2. Polyautoimmunity in CVID

Remarkably, we observed a high percentage of CVID patients with more than one autoimmune disease. According to the literature, polyautoimmunity is defined as the presence of more than one autoimmune disease in a single patient [10,11]. In our series, 15 patients (37% of patients with autoimmunity and 18% of all CVID population) showed two or more autoimmune diseases, indicating the presence of polyautoimmunity. As in the group with monoautoimmunity, even in patients with polyautoimmunity, cytopenias were the most common manifestation. Conversely, enteropathy was recorded only in patients with polyautoimmunity (27%, *p* = 0.006). Moreover, patients with polyautoimmunity showed a significantly lower mean age at diagnosis (−12 years, *p* = 0.018) compared to those with monoautoimmunity. The main result of our study was that diagnostic (+5.6 years) and therapeutic (+7.2 years) delay (*p* = 0.093 and 0.054, respectively) were longer in CVID patients with autoimmunity. An early start of Ig replacement therapy with the reduction of infections and the subsequent chronic antigen stimulation and impaired immune response may decrease the emergence of autoimmune complications. Even if our data have to be proven in larger studies, Ig replacement therapy can be started as soon as possible to prevent severe organ damage and CVID autoimmune complications.

### 4.3. Autoantibodies in CVID Patients

Despite the hypogammaglobulinemia and the defect in specific antibody responses, we identified several types of autoantibodies, mainly anti-TPO antibodies (*n* = 8) and ANA (*n* = 5). In most cases, the autoantibody correlated with the main autoimmune disease. In other patients, we did not find any correlation between the autoantibody pattern and the underlying disease: one subject with anti-TPO antibodies has not developed autoimmune thyroiditis yet, and one patient with LAC positivity had enteropathy. These data raise the question of the meaning of autoantibody positivity in the absence of a definite autoimmune disease in CVID patients as in the general population. Low-intensity ANAs are present in 15% up to 40% of healthy individuals [25], so cut-off titers are used to exclude most of these low-intensity, clinically not-relevant findings. However, at least 5% of the healthy population has a moderate ANA titer that is considered positive [25,26], with relatively higher rates in women and the elderly [15]. We do not know the diagnostic significance of a positive autoantibody that does not correlate with a specific disease. Positive ANA must always be correlated with the clinic [27,28]. In classical autoimmune diseases that are not correlated to immune deficiency, autoantibodies are helpful markers not only for the diagnosis and the classification of the disease but also for monitoring specific tissue/organ damage. For example, anti-dsDNA antibodies and anti-citrullinated peptides antibodies (ACPA) are specific for systemic lupus erythematosus (SLE) and rheumatoid arthritis (RA), respectively [29,30]. Combinations of specific antibodies are also predictive of the possible evolution of undifferentiated clinical variants at the beginning of their presentation. A previous study found that ANA, anti-La/SSB, anti-Ro/SSA, and antiphospholipid antibodies appeared 3.4 years before SLE diagnosis [31]. In rheumatoid arthritis, it was demonstrated that the presence of IgG or IgA ACPA preceded the disease’s onset by years [32]. CVID patients affected by latent polyautoimmunity can develop an overt autoimmune disease over time, depending on several factors, including the pathogenicity of autoantibodies and genetic, epigenetic, and environmental factors [19].

### 4.4. Treatment Modalities in CVID Patients

Regarding the treatment of autoimmune complications in CVID patients, we prescribed disease-modifying antirheumatic drugs (methotrexate), immunosuppressants (including rituximab), and/or biologic agents (adalimumab) in addition to Ig replacement therapy. Except for the patient with autoimmune hepatitis who stopped taking azathioprine because of gastrointestinal intolerance, all other patients tolerated these treatments well.

High-dose IVIg (2 g/kg monthly) and 20%SCIg have been employed as an immunomodulatory agent in patients with autoimmune diseases not linked to CVID [33,34,35,36] and were demonstrated to be beneficial and to have an effective steroid-sparing effect [37,38], thus reducing the infectious risk linked to chronic immunosuppressive treatment. It is possible that even medium- or low-dose Ig protocols (from 0.4 to 1 g/kg/month by intravenous or subcutaneous route) can have immunoregulatory properties, acting on different targets, humoral and/or cellular, including inflammatory cells, regulatory T, B, and NK cells [39,40,41].

### 4.5. Immune Dysregulation in CVID Patients with Autoimmune Diseases and Polyautoimmunity

Several hypotheses have been proposed to explain how autoimmune disorders can develop in patients with a compromised immune system lacking the capacity to mount an effective humoral response. One theory refers to the role of germinal centers, which develop in secondary lymphoid organs during the immune response to foreign antigens [42]. In the germinal centers, there is an equilibrium between the need to organize a protective humoral response with specific antibody affinity and the risk of stimulating an abnormal response to self-components. This complex system is regulated by several lymphoid cell populations, including FOXP3+ T follicular regulatory cells, a T regulatory subset that guarantees the production of specific antibodies while preventing (or reducing) an abnormal autoreactive response [43]. An impairment in humoral response is responsible for antibody immunodeficiency or inadequate vaccine responses. On the opposite, the surge of autoreactivity could lead to autoimmune disorders characterized by excessive autoantibody production, such as SLE and Sjogren’s syndrome, or by the presence of specific autoantibodies (such as anti-acetylcholine receptor antibodies in Myasthenia gravis).

We acknowledge the limitations of our study, as it is observational in a single-center context and small in population. A limitation of our study was the inability to evaluate B cell subpopulations (classical naïve (CD19+CD27−IgD+), switched memory (CD19+CD27+IgD−) and unswitched (CD19+CD27+IgD+)) in all patients but only in 31 patients (38%) as this study was available only after 2017. Therefore, it was not possible to distinguish all patients of our series according to the Freiburg classification [44]. Moreover, the small number of patients studied can affect the statistical power. It is likely that by expanding the sample size, we could increase the power of the statistical analyses.

## 5. Conclusions

Autoimmune diseases constitute the most common non-infectious complication in CVID patients, leading to a significant impact on the quality of life, morbidity, and increased mortality [45]. Since autoimmune manifestations can precede the diagnosis of CVID by several years, it is important to evaluate serum protein electrophoresis and serum Ig (IgG, IgA, IgM, IgE) levels at the diagnosis of an autoimmune disease to early detect a possible hypogammaglobulinemia. Furthermore, it is correct to require serum electrophoresis and serum levels of Ig in cases of neoplasms or other non-infectious CVID-associated complications, such as granulomatosis and enteropathy [46,47]. CVID should be suspected mainly in patients with lymphoma, autoimmune disorders such as ITP, autoimmune hemolytic anemia, and gastrointestinal diseases, including inflammatory bowel diseases [46,47].

Our study highlights the complexity of the CVID phenotypic spectrum secondary to a multi-level dysregulation of the immune system, leading to a significant diagnostic delay due to a missed recognition during early symptoms. Polyautoimmunity during CVID is still little known, and our data highlight the need for larger, more comprehensive investigations guided by artificial intelligence [48] to better characterize the clinical and laboratory features of these patients and to better understand the role of autoantibodies in latent autoimmunity.

## Figures and Tables

**Table 1:** Baseline characteristics of CVID patients (*n* = 81) and clinical phenotypes according to Chapel et al. [10].

Gender: Female	*n* (%)	54 (66.6%)
Age at first clinical presentation (years)	Mean ± SD ^1^	32 ± 21
Age at CVID diagnosis (years)	Mean ± SD ^1^	44 ± 18
Diagnostic delay (years)	Mean ± SD ^1^	11 ± 14
Therapeutic delay (years)	Mean ± SD ^1^	11 ± 14
Mean serum IgG levels at diagnosis (mg/dl)	Median [IQR] ^2^	310 [10–484]
Mean serum IgA levels at diagnosis (mg/dl)	Median [IQR] ^2^	32 [9–64]
Mean serum IgM levels at diagnosis (mg/dl)	Median [IQR] ^2^	31 [17–69]
No other disease-related complications (“infections only”)	*n* (%)	21 (26%)
Autoimmunity	*n* (%)	40 (49%)
Polyclonal lymphoproliferation	*n* (%)	24 (29%)
Enteropathy	*n* (%)	13 (16%)
Malignancy	*n* (%)	21 (26%)
Ig replacement therapy IVIg 20%SCIg fSCIg	*n* (%)	74 (92%)
		28 (35%)
		20(25%)
		26 (32%)
Antibiotic prophylaxis	*n* (%)	5 (6%)
Other treatments Glucocorticoids Methotrexate/Azathioprine Rituximab Adalimumab	*n* (%)	
		14 (17%)
		2 (2.4%)/1 (1.2%)
		1 (1.2%)
		2 (2.4%)
Deceased patients	*n* (%)	7 (9%)

^1^ SD: standard deviation. ^2^ IQR: interquartile range. Diagnostic delay is defined as the time between the occurrence of the first CVID symptom (e.g., infection recurrences, ITP, neoplasia) and the effective CVID diagnosis. Therapeutic delay is defined as the time between the occurrence of the first symptom and the initiation of Ig replacement therapy.

**Table 2:** Main characteristics of CVID patients with (*n* = 40) and without autoimmunity (*n* = 41).

Baseline Characteristics		Patients with Autoimmune Disease (*n* = 40)	Patients Without Autoimmune Disease (*n* = 41)	*p*-Value
Age at CVID diagnosis (years)	Mean ± SD ^1^	44 ± 18	44 ± 19	0.877
Gender (Females)	*n* (%)	26 (72.2%)	28 (62.2%)	0.343
Mean serum IgG levels at diagnosis (mg/dl)	Median [IQR] ^2^	401 [287–485]	412 [269–519]	0.959
Diagnostic delay ^3^ (years)	Mean ± SD ^1^	16.4 ± 18.7	10.8 ± 13.6	0.093
Age of diagnosis: 10–40 years old		8.7 ± 9.9 *	8.7 ± 10.0	1.000
Age of diagnosis: >40–60 years old		27.4 ± 19.1 *	16.7 ± 18.4	0.161
Age of diagnosis: >60 years old		10.5 ± 21.3 *	5.4 ± 4.7	0.495
Symptoms’ onset: 10–40 years old		22.1 ± 19.5 **	15.1 ± 15.7	0.203
Symptoms’ onset: >40–60 years old		3.0 ± 4.0 **	4.3 ± 5.6	0.645
Symptoms’ onset: >60 years old		0.9 ± 0.7 **	3.8 ± 3.4	0.146
Therapeutic delay ^3^ (years)	Mean ± SD ^1^	18.1 ± 18.9	10.9 ± 13.2	0.054
Age of diagnosis: 10–40 years old		12.0 ± 11.3 °	9.0 ± 9.2	0.502
Age of diagnosis: >40–60 years old		28.1 ± 19.1 °	15.1 ± 18.4	0.096
Age of diagnosis: >60 years old		11.2 ± 22.7 °	4.6 ± 4.8	0.409
Symptoms’ onset: 10–40 years old		24.4 ± 19.4 °°	14.9 ± 15.4	0.098
Symptoms’ onset: >40–60 years old		3.8 ± 4.0 °°	3.0 ± 5.5	0.768
Symptoms’ onset: >60 years old		1.1 ± 0.7 °°	3.9 ± 3.5	0.158
Polyclonal lymphoproliferation	*n* (%)	14 (35%)	10 (24%)	0.296
Enteropathy	*n* (%)	4 (10%)	9 (21%)	0.143
Malignancy	*n* (%)	15 (37%)	6 (14%)	0.019

* *p* = 0.0202, ** *p* = 0.0144, ° *p* = 0.0458, °° *p* = 0.0069. ^1^ SD: standard deviation. ^2^ IQR: interquartile range. ^3^ Diagnostic delay is defined as the time between the occurrence of the first CVID-related symptom (e.g., infection recurrences, ITP, neoplasia) and the effective CVID diagnosis. The therapeutic delay is defined as the time between the occurrence of the first CVID-related symptom and the initiation of Ig replacement therapy.

**Table 3:** Autoimmune diseases, autoantibodies, and autoimmune disease treatments in patients with polyautoimmunity.

	Sex/Age at the Diagnosis of CVID	Features at the Onset	Autoimmune Disease n. 1/Age at Diagnosis	Autoimmune Disease n. 2/Age at Diagnosis	Autoimmune Disease n. 3/Age at Diagnosis	Autoantibodies	Autoimmune Disease Treatment
1	F/30	Autoimmune cytopenias	ITP/30	AIHA/49	No	Neg	Glucocorticoids
2	M/28	Recurrent respiratory infections	Coeliac disease/30	Psoriasis/30	No	IgA anti-endomysium antibodies	
3	M/26	Recurrent respiratory infections	ITP/23	Thyroiditis/23	No	anti-TPO, anti-platelet autoantibodies	
4	F/28	Recurrent respiratory infections	ITP/40	Thyroiditis/52	No	anti-TPO	Glucocorticoids
5	F/75	Recurrent respiratory infections	Vitiligo/ND	Psoriasis/75	Psoriatic arthritis/77	Neg	Glucocorticoids
6	F/27	Recurrent respiratory infections	ITP/8	Psoriatic arthritis/16	No	Neg	Methotrexate
7	F/38	Enteropathy + urinary tract infections	Polyserositis/38	Alopecia/41	No	Neg	Glucocorticoids
8	F/29	Recurrent respiratory infections + urinary tract infections	Hypoparathyroidism/37	ITP/40	No	Neg	Glucocorticoids
9	F/19	Recurrent respiratory infections	Seronegative spondyloarthritis/19	Vitiligo/19	Thyroiditis/20	anti-TPO	Glucocorticoids, Adalimumab
10	F/60	Recurrent respiratory infections	Vitiligo/16	Atrophic gastritis/54	Pernicious anemia/55	Neg	
11	F/52	Recurrent respiratory infections + enteropathy	Seronegative spondyloarthritis/62	Chronic gastritis/63	Primary biliary cholangitis/65	Neg	Glucocorticoids Adalimumab
12	F/24	Recurrent respiratory infections + enteropathy + urinary tract infections	Thyroiditis/46	ITP/48	No	anti-TPO	
13	F/62	Autoimmune diseases	Autoimmune hepatitis/62	Thyroiditis/62	Coeliac disease/62	ANA	
14	M/23	Recurrent respiratory infections	No	No	No	anti-TPO	
15	M/25	Recurrent respiratory infections + enteropathy	No	No	No	LAC	

Abbreviations: ANA = anti-nuclear autoantibodies; anti-TPO, anti-thyroid peroxidase; ITP, immune thrombocytopenia; LAC = lupus anticoagulant. F/M: female/ male; Neg: negative; No: number.

**Table 4:** Main clinical CVID characteristics in patients with polyautoimmunity.

N	Sex/Age at the CVID Diagnosis	Polyautoimmunity	Bronchiectasis	Granulomatosis (site/s)	Epatopathy	Enteropathy	Malignancy/Lymphoproliferative Disorder	Splenomegaly	Other Comorbidities	Ongoing Therapy
1	F/30	ITP+ autoimmune hemolytic anemia	No	Yes (lymph nodes, spleen)	No	No	No	Yes (splenectomy)	No	20%SCIg
2	M/28	Coeliac disease+ psoriasis	No	No	HBV-related	Yes	Mycosis fungoides	No	No	IVIg
3	M/26	ITP + thyroiditis	No	No	No	No	No	No	No	No
4	F/28	ITP + thyroiditis	No	Yes (GLILD, lymph nodes)	HCV fibrosis	Yes	LGL-NK lymphoproliferative disorder	Yes (splenectomy)	No	20%SCIg
5	F/75	Psoriatic arthritis + vitiligo	No	No	No	No	Gastric GIST	No	Wasp anaphylactic shock	IVIg
6	F/27	ITP + Psoriatic arthritis	No	No	No	No	No	No	No	IVIg
7	F/38	Alopecia + polyserositis	No	No	No	Yes	No	No	No	IVIg
8	F/29	ITP + hypoparathyroidism	No	Yes (GLILD, lymph nodes, liver)	Hepatic cirrhosis	No	No	Yes	No	fSCIg
9	F/19	Seronegative spondyloarthritis + vitiligo + thyroiditis	No	No	No	No	No	No	Allergic asthma, epilepsy	fSCIg
10	F/60	Vitiligo+ pernicious anemia	Yes	No	No	No	Gastric adenocarcinoma	No	No	20%SCIg
11	F/52	PBC + seronegative spondyloarthritis + chronic gastritis	Yes	Yes (GLILD, spleen, liver)	PBC	Yes	Melanoma	Yes	No	IVIg
12	F/24	ITP+ thyroiditis	No	Yes (GLILD, lymph nodes)	No	No	Thyroid cancer	No	No	IVIg
13	F/62	Autoimmune hepatitis + coeliac disease + thyroiditis	No	No	No	No	No	Yes	No	fSCIg
14	M/23	No	No	No	No	No	No	No	Chronic spontaneous urticaria	IVIg
15	M/25	No	No	No	Non-alcoholic hepatic steatosis	Yes	No	No		fSCIg

Abbreviation: GIST, gastrointestinal stromal tumor; GLILD, granulomatous and lymphocytic interstitial lung disease; HBV, hepatitis B virus; HCV, hepatitis C virus; ITP, immune thrombocytopenia; LGL/NK, large granular lymphocyte/natural killer; PBC, primary biliary cholangitis. fSCIg: facilitated subcutaneous immunoglobulin; IVIg: intravenous immunoglobulin; 20%SCIg: 20%subcutaneous immunoglobulin.

**Table 5:** Main characteristics of CVID patients with monoautoimmunity (*n* = 25) and polyautoimmunity (*n* = 15).

Baseline Characteristics		All Patients with Autoimmune Disease (*n* = 40)	Patients with Polyautoimmunity (*n* = 15)	Patients with Monoautoimmunity (*n* = 25)	*p*-Value Between Patients with Poly- and Monoautoimmunity
Age at CVID diagnosis (years)	Mean ± SD ^1^	44 ± 18	36 ± 17	48 ± 13	0.018
Mean serum IgG levels at diagnosis (mg/dl)	Median [IQR] ^2^	408 [269–494]	362 [317–505]	422 [265–485]	0.695
Diagnostic delay ^3^ (years)	Mean ± SD ^1^	16.4 ± 18.7	10.9 ± 14.9	19.7 ± 20.2	0.197
Therapeutic delay ^3^ (years)	Mean ± SD ^1^	18.1 ± 18.9	14.5 ± 16.2	20.2 ± 20.5	0.414
Polyclonal lymphoproliferation	*n* (%)	14 (35%)	5 (33%)	9 (36%)	0.864
Enteropathy	*n* (%)	4 (10%)	4 (27%)	0 (0%)	0.006
Malignancy	*n* (%)	15 (38%)	6 (40%)	9 (36%)	0.800

^1^ SD: standard deviation. ^2^ IQR: Interquartile range. ^3^ Diagnostic delay is defined as the time between the occurrence of the first CVID-related symptom (e.g., infection recurrences, ITP, neoplasia) and the effective CVID diagnosis. Therapeutic delay is defined as the time between the occurrence of the first CVID-related symptom and the initiation of Ig replacement therapy.

## Data Availability

Data will be made available on request. The data are those present in the clinical charts of all patients. They are subject to Privacy according to the Italian Laws.

## Supplementary Materials

The following supporting information can be downloaded at: https://www.mdpi.com/article/10.3390/biomedicines13030552/s1, Table S1. Baseline examinations in our series of 81 patients with CVID. Table S2. Immunological parameters employed in the study.

## Abbreviations

ANA	antinuclear antibodies
CVID	common variable immunodeficiency
IEI	inborn errors of immunity
IVIg	intravenous immunoglobulin
SCIg	subcutaneous immunoglobulin
