# Correction: Danieli et al. Polyautoimmunity Reflecting Immune Dysregulation in Common Variable Immunodeficiency. *Biomedicines* 2025, *13*, 552

**DOI:** 10.3390/biomedicines13102396

**Published:** 2025-09-30

**Authors:** Maria Giovanna Danieli, Giuseppe Murdaca, Cristina Mezzanotte, Ilaria Claudi, Elena Buti, Matteo Martini, Maria Beatrice Bilò, Sebastiano Gangemi, Gianluca Moroncini

**Affiliations:** 1 SOS Immunologia delle Malattie Rare e dei Trapianti, SOD Clinica Medica, Dipartimento di Medicina Interna, Azienda Ospedaliero Universitaria delle Marche, 60126 Ancona, Italy; m.g.danieli@univpm.it; 2 Department of Clinical and Molecular Sciences, Marche Polytechnic University, 60126 Ancona, Italy; m.b.bilo@univpm.it (M.B.B.); g.moroncini@univpm.it (G.M.); 3 Postgraduate School in Allergology and Clinical Immunology, Università Politecnica delle Marche, 60126 Ancona, Italy; ilari.claudi@gmail.com (I.C.); elena.buti2017@gmail.com (E.B.); 4 Department of Internal Medicine, University of Genoa, 16132 Genoa, Italy; 5 Allergology and Clinical Immunology Unit, Ospedale San Bartolomeo, 19038 Sarzana, Italy; 6 UO Medicina Interna, Azienda Sanitaria Territoriale Pesaro Urbino, 61032 Fano, Italy; cristina.mezzanotte.1@gmail.com; 7 SOSD Allergologia, Dipartimento di Medicina Interna, Azienda Ospedaliero Universitaria delle Marche, 60126 Ancona, Italy; matteo.martini@ospedaliriuniti.marche.it; 8 Operative Unit of Allergy and Clinical Immunology, Department of Clinical and Experimental Medicine, University of Messina, Via Consolare Valeria 1, 98125 Messina, Italy; sebastiano.gangemi@unime.it; 9 Clinica Medica, Department of Internal Medicine, Marche University Hospital, 60126 Ancona, Italy

## Reference Update

In the original publication [1], the authors unintentionally cited a retracted reference, citation reference 25. The authors have updated the retracted citation reference. The new citation reference appears below. The authors state that the scientific conclusions are unaffected. This correction was approved by the Academic Editor. The original publication has also been updated.

25. Pashnina, I.; Krivolapova, I.; Fedotkina, T.; Ryabkova, V.; Chereshneva, M.; Churilov, L.; Chereshnev, V. Antinuclear Autoantibodies in Health: Autoimmunity Is Not a Synonym of Autoimmune Disease. *Antibodies* **2021**, *10*, 9. https://doi.org/10.3390/antib10010009.

## Citation Error

In the original publication [1], a reference to reference 24 was incorrectly cited as 23. The citation has now been changed in Section 4.1, Paragraph 4, and should read:

Rheumatological manifestations are detected in 11% of all our CVID patients (22.5% of the AD population). In agreement with the literature, rheumatologic diseases are reported in a range from 5.9% [24] to 10% of CVID patients [5], with, as expected, a female preponderance [24]. Psoriatic arthritis and cutaneous psoriasis are relatively frequent in our cohort, probably due to the higher prevalence of these conditions in our Region (3.5% in the Marche Region against 2.8% in Italy).
