# Supplementary material for: Polyautoimmunity Reflecting Immune Dysregulation in Common Variable Immunodeficiency
Source: Biomedicines. 2025 Feb 21;13(3):552. doi: 10.3390/biomedicines13030552 (PMC11940294; doi:10.3390/biomedicines13030552)
Supplement: Supplementary file 1 [file biomedicines-13-00552-s001.zip › biomedicines-3483161-supplementary.pdf]

Data Collection and Evaluation

Comprehensive data collection was performed for each patient, including demographic information and a detailed medical history. This encompassed date of birth and death (reporting the cause of death), age onset of symptoms, clinical manifestations at diagnosis and during follow-up, comorbidities, autoantibodies and laboratory blood tests, concurrent medications, family history of immunodeficiencies and autoimmune diseases, consanguinity among parents and grandparents, and history of malignancy and therapy.

Our patients underwent regular clinical follow-up every 3–6 months. This follow-up included a comprehensive physical examination and a detailed medical history, with a particular focus on recurrent infections and the development or progression of disease complications.

At the first visit, routine analyses and specific exams aimed at confirming the diagnosis of CVID and excluding secondary immunodeficiencies were performed according to validated techniques (Tables S1 and S2). As for immunological parameters, we considered the assay positive if the ANA titer was ≥1:160. We assessed ANA before the introduction of Ig therapy. The ANA tests were repeated (at least 3 to 4 weeks after the administration of IVIg therapy) during follow-up in case of signs suggestive of autoimmune disease.
Depending on the clinical manifestations, the following parameters were also assessed in selected patients: IgM rheumatoid factor, anti-cyclic citrullinated peptide (anti-CCP) antibodies, anti-double stranded (ds)-DNA antibodies, anti-extractable nuclear antigen (anti-ENA) antibodies with immunoblotting analysis to identify different patterns, anti-neutrophil cytoplasmic antibodies (ANCA), and anti-transglutaminase and anti-endomysium antibodies (Table 2). Finally, we completed the study of suspected coeliac disease, searching for HLA-DQ2 and DQ8 haplotypes.

Additional examinations were conducted to gather more comprehensive information about the patients' health status and to evaluate specific organ systems and potential complications associated with CVID (Table S2).

The timing for follow-up was based on the clinical conditions and serum Ig values. Generally, the patients were tested for Ig levels and routine analyses every 3–6 months and every 12–24 months for the exclusion of emerging causes of secondary immunodeficiency. Pulmonary function tests, abdominal ultrasounds, UBT, or H. pylori stool antigen studies were repeated annually in all cases. The repetition time of other laboratory and/or instrumental examinations was defined according to the clinical condition of the patient. For gastroscopy, we recommend strict monitoring in CVID patients who present one of the following risk factors: age ≥50; smoking history; family history of gastric carcinoma; upper gastrointestinal symptoms; previous gastroscopy with inadequate biopsies or finding of active gastritis, dysplasia, atrophic gastritis, or H. pylori detection; and iron or vitamin B12 deficiency. As a screening test, we performed a colonoscopy every 5 years [6].

Treatment

In our center, patients were treated and monitored based on the prevailing clinical practices at the time of their management. Immunoglobulin replacement therapy (Ig-RT) represents a lifesaving therapy in antibody deficiencies. Patients received Ig replacement therapy using intravenous Ig (IVIg), subcutaneous

IG (20%SCIg, conventional), and facilitated SCIg (fSCIg). Ig can be administered intravenously (IVIg) or subcutaneously (SCIg, conventional or by manual push, and facilitated SCIg). Intravenous administration achieves a rapid high peak plasma concentration of IgG, providing immediate protection against infections. Conventional subcutaneous administration is a viable alternative because it can be performed at home; the infusion site is the subcutaneous tissue, leading to a slower absorption rate and smaller infused volumes, but serum IgG levels remain stable for a longer period. A further innovation is the facilitated subcutaneous administration, which extends the dosing interval to 3-4 weeks by preceding the IgG injection with the hyaluronidase, allowing the administration of larger drug volumes.

The monthly dose of Ig administered ranged from 0.4 to 0.6 g/kg according to the procedures previously described [15,39]. The dosage was adjusted based on factors such as recurrent infections and serum IgG levels. For patients with contraindications to Ig therapy or those who did not respond adequately to Ig treatment, antibiotic prophylaxis was implemented. In cases of autoimmune complications, treatment approaches included the use of glucocorticoids, hydroxychloroquine, immunosuppressants, and biological drugs. The choice of therapy was based on the individual patient's needs and the specific autoimmune complications reported.

Table S1. Baseline examinations in our series of 81 patients with CVID.

**Routine analyses**	Complete blood count<br>Liver enzymes<br>Renal function indexes<br>Serum electrophoresis<br>Quantitative detection of serum protein and albumin<br>C reactive protein (CRP)<br>Erythrocyte sedimentation rate (ESR)<br>Urine analysis
**Immunological tests**	Serum IgG, IgA, IgM, IgE and IgG subclasses levels<br>Serum and urine immunofixation<br>Serum and urine-free light Ig chains<br>Antinuclear antibodies (ANA)<br>Anti-phospholipid antibodies (anticardiolipin, anti-beta2 glycoprotein-I antibodies) and Lupus anticoagulant (LAC)<br>C3 and C4 levels<br>Direct antiglobulin test (DAT)<br>Lactate dehydrogenase and beta-2 microglobulin<br>Thyroid-stimulating hormone (TSH) levels; anti-thyroid peroxidase (anti-TPO) antibodies<br>Flow-cytometric analysis of peripheral lymphocyte
**Infectious disease testing**	HCV RNA<br>HIV RNA<br>HBV DNA
**Specific laboratory tests (based on clinical manifestations)**	IgM rheumatoid factor<br>Anti-cyclic citrullinated peptide (anti-CCP) antibodies<br>Anti-double stranded (ds)-DNA antibodies<br>Anti-extractable nuclear antigen (anti-ENA) antibodies with immunoblotting analysis to identify different patterns<br>Anti-neutrophil cytoplasmic antibodies (ANCA)<br>Anti-transglutaminase and anti-endomysium antibodies<br>HLA-DQ2 and DQ8 haplotypes<br>Fecal calprotectin<br>Helicobacter pylori stool antigen research
**Instrumental tests**	Hepato-splenic and abdominal ultrasounds<br>Pulmonary function tests and evaluation of diffusing capacity of the lung for carbon monoxide (DLCO)<br>Chest and paranasal sinus computed tomography (CT) scan<br>Upper endoscopy<br>Colonoscopy

Table S2. Immunological parameters employed in the study.

Test	Method	Reference values
IgG	Turbidimetric immunoassay	650-1600 mg/dl (≥10 years)
IgA	Turbidimetric immunoassay	40-350 mg/dl (≥10 years)
IgM	Turbidimetric immunoassay	50-300 mg/dl (≥10 years)
Anti- CCP	CLIA	Positive ≥20 IU/ml
Anti-dsDNA	CLIA	Negative <27 IU/ml<br>Borderline 27-35 IU/ml<br>Positive >35 IU/ml
p-ANCA/c-ANCA	CLIA	Positive >6 IU/ml
Anti-cardiolipin (aCL) IgG	CLIA	Positive if >20 CU
Anti-cardiolipin (aCL) IgM	CLIA	Negative ≤9,9 CU<br>Positive >10 CU
Lupus anticoagulant (LAC)	SCT<br>DRVVT	Ratio normalized 0,89<br>Ratio normalized 1,10
Anti-beta2GPI IgG	CLIA	Negative ≤20 CU
Anti-beta2GPI IgM	CLIA	Negative ≤20 CU
C3	Turbidimetric immunoassay	84-160 mg/dl
C4	Turbidimetric immunoassay	12-36 mg/dl
Direct antiglobulin testing<br>Indirect antiglobulin testing	Observation of agglutination	Negative<br>Absent
Anti-transglutaminase IgG	FEIA	Negative ≤10 U/ml
Anti-transglutaminase IgA	FEIA	Negative ≤10 U/ml
Anti-endomysium antibodies IgA	IFI	Negative

Abbreviations: IFI=indirect immunofluorescence; CLIA= chemiluminescence immunoassay; FEIA= fluorometric enzyme-linked immunoassay; CU= conventional unit; IU= international unit; DRVVT= dilute Russell's viper venom time; SCT= silica clotting time.
